# Characterization of duckweed-associated bacteria from the *Wolffia globosa* microbiome: Strain-level effects on host growth and key determinants

**DOI:** 10.3934/microbiol.2026022

**Published:** 2026-07-30

**Authors:** Yuparat Saimee, Ryusuke Okada, Daisuke Inoue, Michihiko Ike

**Affiliations:** Division of Sustainable Energy and Environmental Engineering, The University of Osaka, 2-1 Yamadaoka, Suita, Osaka 565-0871, Japan

**Keywords:** *Wolffia* growth, duckweed-associated bacteria, plant growth-promoting bacteria, plant growth-inhibiting bacteria, plant growth-promoting traits

## Abstract

*Wolffia*, a rootless duckweed in the family Lemnaceae, is increasingly recognized as a sustainable bioresource for food, animal feed, bioenergy, and biomaterials. Its productivity depends strongly on the associated microbiome, yet the functional roles of individual duckweed-associated bacteria (DAB) remain poorly understood. In this study, we evaluated the effects of DAB isolated from *Wolffia globosa* microbiomes on host plant growth and characterized their physiological traits. A total of 98 DAB strains were isolated from seven *W. globosa* microbiomes and individually co-cultivated with *W. globosa*, exhibiting diverse effects on host growth ranging from promotion to inhibition, with −14.6 to +12.9% of final effects on plant growth. Ten strains consistently promoted host growth and nine strains inhibited growth, which were identified as plant growth-promoting and growth-inhibiting bacteria, respectively. Of these, seven and four strains were identified within the core taxa of *W. globosa* microbiomes. At the community level, growth-promoting strains tended to be enriched in the growth-promoting microbiomes, whereas neutral or negative strains appeared to be enriched in the growth-inhibiting microbiomes. Taxonomic evaluation demonstrated that prominent promotive and inhibitory phenotypes occurred across six and five genera, respectively, with strain-level characteristics likely determining plant growth effects. Indole-related compound production by DAB emerged as a key physiological determinant associated with *Wolffia* growth. These findings provide a traceable strain set to advance our understanding of *Wolffia*–microbiome interactions and offer practical clues on microbiome engineering strategies to enhance *Wolffia* biomass productivity.

## 1. Introduction

*Wolffia* is a rootless genus within the family Lemnaceae (commonly known as duckweed) and represents the smallest and fastest-growing plant among higher plants [1]. This genus is found across tropical and temperate regions and thrives in various aquatic habitats, including freshwater and wastewater, under favorable conditions [2]–[4]. *Wolffia* has emerged as a valuable bioresource for human consumption and animal feed, as well as a promising feedstock for bioenergy and biomaterial production. This is attributed to its exceptionally rapid proliferation and protein-rich composition, which includes all essential amino acids, favorable dietary nutritional profiles, and bioavailable vitamin B12 [5]–[9].

The biomass productivity and nutritional value of *Wolffia*, similar to those of rooted duckweeds and other plants, are strongly influenced by the associated microbiome [10],[11]. Within the microbiome, bacteria, namely duckweed-associated bacteria (DAB), directly interact with the host in mutualistic, commensal, or antagonistic interactions. DAB that benefit duckweed growth through multiple mechanisms, such as phosphate solubilization, siderophore production, and phytohormone synthesis, are known as plant growth-promoting bacteria (PGPB). In contrast, certain DAB suppress duckweed growth without inducing noticeable pathogenic symptoms, known as plant growth-inhibiting bacteria (PGIB). Several DAB, including *Acinetobacter calcoaceticus* P23 [12]–[14] and *Aquitalea magnusonii* H3 as PGPB and *Acinetobacter ursingii* M3 and *Asticcacaulis excentricus* M6 as PGIB [15], have been isolated and characterized for their interactions with rooted duckweeds. In addition to single DAB, studies on natural and synthetic communities have also been characterized for the interactions between microbe-microbe and duckweed-microbe and can substantially influence duckweed growth, stress resilience, and host-microbe mutualistic outcomes [16],[17]. However, such duckweed-microbiome interactions have been extensively examined in rooted duckweeds, particularly, *Lemna minor* [12]–[17]. Given the leaf-like structure of *Wolffia*, it likely differs fundamentally from rooted duckweeds in ecological and physiological aspects. Consequently, interactions between host and microbe may also differ fundamentally due to the release of exudates, metabolites, and other chemical signals [18]. Understanding *Wolffia*–microbiome interactions is therefore crucial for incorporating microbiome-based approaches into *Wolffia* cultivation strategies, given the growing importance of this genus as a food crop and sustainable bioenergy feedstock.

Research has revealed that rootless duckweeds assemble microbiomes distinct from those of rooted duckweeds [19],[20]. In our other study, microbiomes assembled on *Wolffia globosa* from various microbial sources (e.g., freshwater ponds, secondary effluents, and activated sludge) were systematically characterized using 16S rRNA amplicon sequencing [10]. The assembled microbiomes differed in composition and effects primarily depending on the microbial source used and significantly influenced host growth, ranging from inhibition (up to −12.2% frond area) to promotion (up to +55.6% frond area). Although the microbiome composition differed across inoculum sources, seven bacterial families, including *Beijerinckiaceae*, *Caulobacteraceae*, *Comamonadaceae*, *Methylophilaceae*, *Rhizobiaceae*, and *Sphingomonadaceae*, were consistently conserved across all microbial sources and identified as core taxa in *W. globosa* microbiomes [10]. Despite these advances, most studies remain limited to community-level analyses, resulting in a lack of clarity regarding the functional contributions of individual strains of DAB within the microbiomes and limiting the development of reproducible microbiome-based strategies for *Wolffia* production. To address this gap, cultured DAB and strain-level culture-based studies that directly link bacterial identity, functional traits, and host responses are necessary.

Therefore, we aimed to evaluate the effects of DAB from *W. globosa* on host plant growth and characterize the physiological traits that may be associated with this growth. To this end, approximately 100 strains were isolated from *Wolffia* microbiomes established in another study [10]. In this study, taxonomic identity is integrated with functional traits and growth effects for isolated DAB, thereby offering insights into plant–microbiome interactions in rootless duckweeds at both the community and strain levels for the first time. In addition, this study highlights the coexistence of PGPB and PGIB within the *Wolffia* microbiome. The insights gained are anticipated to inform strategies for developing robust *Wolffia* holobionts aimed at enhancing biomass productivity and mitigating adverse effects in practical applications.

## 2. Materials and methods

### 2.1. Isolation of duckweed-associated bacteria

DAB were isolated from 7 of the 18 *W. globosa* microbiomes obtained in another study [10]: 4 (PW_A, PW_B, PW_F, and PW_H), 1 (SE_2), and 2 (AS_1 and AS_2) DAB originated from co-cultivation with bacterial communities derived from freshwater ponds, secondary effluents, and activated sludge, respectively. Among them, PW_A, SE_2, AS_1, and AS_2 promoted *W. globosa* growth at the community level in the previous investigation, and were thus classified as growth-promoting microbiomes in this study. Conversely, PW_B had a neutral effect (neutral microbiome), and PW_F and PW_H exhibited inhibitory effects (growth-inhibiting microbiomes). To isolate DAB, the plants collected after 10 days of co-cultivation in the other study were placed in sterile microtubes containing 200 µL of modified Hoagland (MH) medium [21] and homogenized using a BioMasher II (Nippi, Japan). The homogenates were serially diluted, and 100 µL of each dilution was spread onto R2A agar (DAIGO, Shiotani M.S. Co., Ltd., Japan). The plates were incubated at 28 °C for 3–7 days. All morphologically distinct colonies from each sample were isolated and purified in the same medium. The isolates were routinely maintained on the same medium and stored in R2A broth containing 25% glycerol at −80 °C for long-term preservation. Unless otherwise noted, all cultivations were performed at 28 °C, with rotary shaking at 120 rpm in liquid medium.

### 2.2. Taxonomic identification

Genomic DNA of isolated bacteria was extracted using the Cica Geneus DNA extraction reagent (Kanto Chemical, Japan). The genomic DNA was amplified to obtain partial sequences of the 16S rRNA gene using SapphireAmp Fast PCR Master Mix (Takara Bio Inc., Japan) and universal bacterial primers 27F (5′-AGAGTTTGATCMTGGCTCAG-3′) and 1492R (5′-TACGGYTACCTTGTTACGACTT-3′) in a Bio-Rad T100 Thermal Cycler (Bio-Rad Laboratories, USA). PCR amplification was performed with the following thermocycling conditions: Initial denaturation at 94 °C for 5 min followed by 30 cycles of denaturation at 94 °C for 1 min, annealing at 52 °C for 1 min, and extension at 72 °C for 1 min, and a final extension at 72 °C for 5 min. PCR products were purified via a NucleoSpin Gel and PCR Clean-up Kit (Macherey-Nagel, Germany) and sequenced using an Applied Biosystems 3730xl DNA analyzer (Macrogen Japan Corp., Japan). The obtained 16S rRNA gene sequences were compared with type strain sequences in the EzBioCloud database (https://www.ezbiocloud.net/) [22] for taxonomic identification. A phylogenetic tree was constructed using the neighbor-joining method alongside the maximum composite likelihood model in MEGA11 [23]. The 16S rRNA gene sequences of the isolated DAB were deposited in the GenBank/EMBL/DDBJ databases under accession numbers LC896431–LC896528.

### 2.3. Co-cultivation experiment to assess the effects on Wolffia globosa growth

Prior to the experiments, a loopful of each isolated strain grown on R2A agar was inoculated into a glass vial containing 20 mL of R2A broth and cultured for 24 h. Bacterial cells were harvested by centrifugation (10,000× *g*, 4 °C, 10 min), washed twice with MH medium, and then re-suspended in the same medium to obtain an optical density at 600 nm (OD_600_) of 0.1, measured using a UV-Vis spectrophotometer (UV-1850, Shimadzu, Japan).

Moreover, axenic *W. globosa* was pre-cultivated in MH medium for 5 days under light conditions with an irradiance of 80 µmol/m^2^/s and a photoperiod of 16 h light/8 h dark. Plant sterility was routinely confirmed by the absence of microbial growth on R2A agar after plating *W. globosa* with and without homogenization. It should be noted that this confirmation was limited to culturable bacteria on the applied medium.

The effect of isolated strains on *Wolffia* growth was evaluated using a two-step co-cultivation experiment as described in another study [10]. Twenty fronds of *W. globosa* were transferred to a 6-well plate containing 10 mL of the bacterial inoculum suspension to initiate the co-cultivation experiments. The co-culture was maintained under the aforementioned light conditions for 3 days (colonization stage) to facilitate bacterial attachment to the plant. Subsequently, all the initial 20 fronds and newly proliferated fronds were transferred to a new 6-well plate containing 10 mL of fresh bacteria-free medium and cultivated under identical conditions for 3 days (growing stage) to evaluate the effects of DAB after the attachment process. A control experiment was conducted using axenic *W. globosa* cultivated in sterile MH medium devoid of bacterial inoculum. Co-cultivation experiments involving each isolate, along with control experiments, were performed in quintuplicate.

The growth of *Wolffia globosa* was assessed by measuring the frond area using photographs captured on days 0, 3, and 6. Frond area was quantified using an image processing procedure based on RGB-to-HSV color conversion, which was performed using Python on Google Colab (https://colab.research.google.com), as described previously [10]. The image processing workflow is presented in Supplementary Text 1. The detected frond area data were used to calculate the relative growth rate (RGR), with RGR at day 3 being calculated from days 0 to 3 and RGR at day 6 being calculated from days 0 to day 6, according to Eq 1. Subsequently, RGR values were converted to an effect on plant growth (EPG) value using Eq 2 to assess the impact of isolated bacteria on plant growth. Based on the EPG values, effects were categorized as follows: Values greater than +5% as promotive effects, values between −5% and +5% as neutral effects, and values less than −5% as inhibitory effects [10],[24].

$$RGR=\frac{\ln X_{tn}-\ln X_{t0}}{t_{n}-t_{0}}$$ (1)

where *X_tn_* is the frond area at time *t_n_* (3 and 6 days), *X_t0_* is the initial frond area of *Wolffia* at day 0, *t_n_* represents the time points during the experiment (3 and 6 days), and *t_0_* represents the initial time point (0 days).

$$EPG\left(\%\right)=\frac{G\left(T\right)-G\left(C\right)}{G\left(C\right)}\times 100$$ (2)

where *G(T)* is the mean RGR derived from the *Wolffia* frond area during co-cultivation with a bacterial isolate after 3 and 6 days of cultivation, while *G(C)* is that of the axenic *Wolffia* used as the control. Here, the standard errors (SE) for EPG was calculated using Eq 3.

$$SE\left(EPG\right)=\frac{\sqrt{\left(SE{\left(G\left(T\right)\right)}^{2}+SE{\left(G\left(C\right)\right)}^{2}\right)}}{G(C)}\times 100$$ (3)

where *SE(G(T))* and *SE(G(C))* are the standard deviations of *G(T)* and *G(C)*, respectively.

### 2.4. Physiological characterization

All DAB strains were tested for physiological characteristics associated with plant growth promotion, including the production of siderophore, indole-related compounds (IRCs), and hydrogen cyanide (HCN) as well as phosphate solubilization. The cells from isolated strains, precultured as described above, were re-suspended in R2A broth to achieve an OD_600_ of 1. Subsequently, 10 µL of the cell suspension was spot-inoculated onto each assay medium.

Siderophore production was evaluated on chrome azurol S (CAS) agar medium for 7 days; a change in the halo zone surrounding the colony from blue to yellow indicated positive activity. IRC production was assessed using colorimetric detection [25], with minor modifications. The assay was performed in R2A broth supplemented with 0.05% (w/v) L-tryptophan for 5 days in the dark. Subsequently, the culture was centrifuged (10,000× *g*, 4 °C, 10 min), and 100 µL of the supernatant was mixed with 200 µL of Salkowski reagent. Following a 30-min incubation at room temperature, with air conditioning maintained at 25 °C and in the dark, the absorbance at 530 nm was measured to determine IRC concentrations using indole-3-acetic acid (IAA) as the standard reference. A full-spectrum scan (440–600 nm, 1-nm intervals) was conducted to determine the maximum absorbance and classify specific IRCs based on the specificity of the Salkowski reagent, as outlined by Gilbert et al. [25]. HCN production was assayed using a picrate strip for 1–2 days according to the methodology described by Saber et al. [26]. The phosphate solubilization activity was evaluated using Pikovskaya's agar medium for 7 days. Positive activity was observed in a halo zone below or around the colonies.

### 2.5. Data and statistical analysis

To validate the consistency between culturable DAB and the core microbiome of *W. globosa*, the 16S rRNA gene sequences of isolated DAB were compared with those of amplicon sequence variants (ASVs) of the core *Wolffia* microbiome (BioProject accession number PRJDB18855) obtained from another study [10] using pairwise distance analysis in MEGA11. Strains exhibiting >99% similarity with core ASVs were deemed reliable representatives of core taxa within the *W. globosa* microbiome at the genus level [27].

To assess the relationship between the physiological traits of isolated DAB and *W. globosa* growth, multiple linear regression was performed using the lm() function in R (version 4.3.2). First, a model was fitted using EPG values as the response variable, with phosphate solubilization, siderophore production, HCN production, and IRC production (qualitative data: 1 = positive, 0 = negative) as explanatory variables. To further determine which IRC type contributed to the effect of IRC production, a second model was fitted using IRC type (pink, orange, or brown) as explanatory variable, with strains producing no IRCs serving as the reference. A one-way ANOVA followed by Tukey's HSD post-hoc test was used for comparisons among experimental groups. All statistical significance was set at *p* < 0.05.

## 3. Results and discussion

### 3.1. Isolation and identification of DAB

A total of 98 bacterial strains were isolated from *W. globosa* microbiomes after 10 days of co-cultivation with 7 distinct microbial sources. The number of strains isolated from each community is summarized in Table S1. Of these, 70 strains were isolated from four growth-promoting microbiomes (PW_A, SE_2, AS_1, and AS_2), 9 strains from a neutral microbiome (PW_B), and 19 strains from two growth-inhibiting microbiomes (PW_F and PW_H).

A phylogenetic tree illustrating the evolutionary relationships was constructed for 98 isolated DAB from all microbiome phenotypes (Figure 1a). Although some DAB were assigned to the same species and isolated from the same community, all were treated as distinct strains because of their distinct colony morphologies and further evidence of separated lineages in phylogeny (Figure 1a). The 98 DAB strains were assigned to three phyla: Pseudomonadota (formerly Proteobacteria; 96 strains), Bacillota (formerly Firmicutes; 1 strain), and Actinomycetota (1 strain). At the family level, 17 families were represented. The most dominant family was *Boseaceae* (31/96; classified as *Beijerinckiaceae* in the SILVA SSU v.138.2 database [10]), followed by *Rhizobiaceae* (16/96), *Pseudomonadaceae* (11/96), *Methylobacteriaceae* (10/96; classified as *Beijerinckiaceae* in the SILVA SSU v.138.2 [10]), and *Sphingomonadaceae* (10/96) (Figure 1b). The predominance of Proteobacteria among the isolated DAB was consistent with previous reports, in which this phylum has been repeatedly reported as the primary associate of duckweed, including *Wolffia* [10],[19],[20],[28],[29]. *Boseaceae*, *Rhizobiaceae*, and *Sphingomonadaceae* dominated the growth-promoting microbiomes (PW_A, SE_2, AS_1, and AS_2), whereas *Pseudomonadaceae* predominated in the growth-inhibiting microbiomes (PW_F and PW_H). This pattern agrees with the relative abundance reported in a study based on 16S rRNA amplicon sequencing [10]. Although members of *Boseaceae* were isolated from all communities except PW_F, they were detected in the PW_F microbiome data [10]. This family has been proposed as a potential key player promoting *W. globosa* growth and as a unique core taxon of *W. globosa* microbiomes [10]. Taken together with the other study, these findings emphasize *Boseaceae* (or *Beijerinckiaceae*) as a key lineage closely associated with *W. globosa* and a characteristic core taxon of its microbiome.

At the genus level, the isolated DAB represented 29 genera across all microbiome phenotypes, dominated by *Bosea*, *Rhizobium*, *Pseudomonas*, *Methylorubrum*, and *Sphingopyxis* (Figure 1b). *Bosea* and *Pseudomonas* appeared across all phenotypes; *Bosea* dominated the growth-promoting and neutral microbiomes, whereas *Pseudomonas* dominated growth-inhibiting microbiomes. *Rhizobium* occurred in growth-promoting and growth-inhibiting microbiomes, whereas other dominant genera were restricted to the growth-promoting communities.

**Figure 1.**
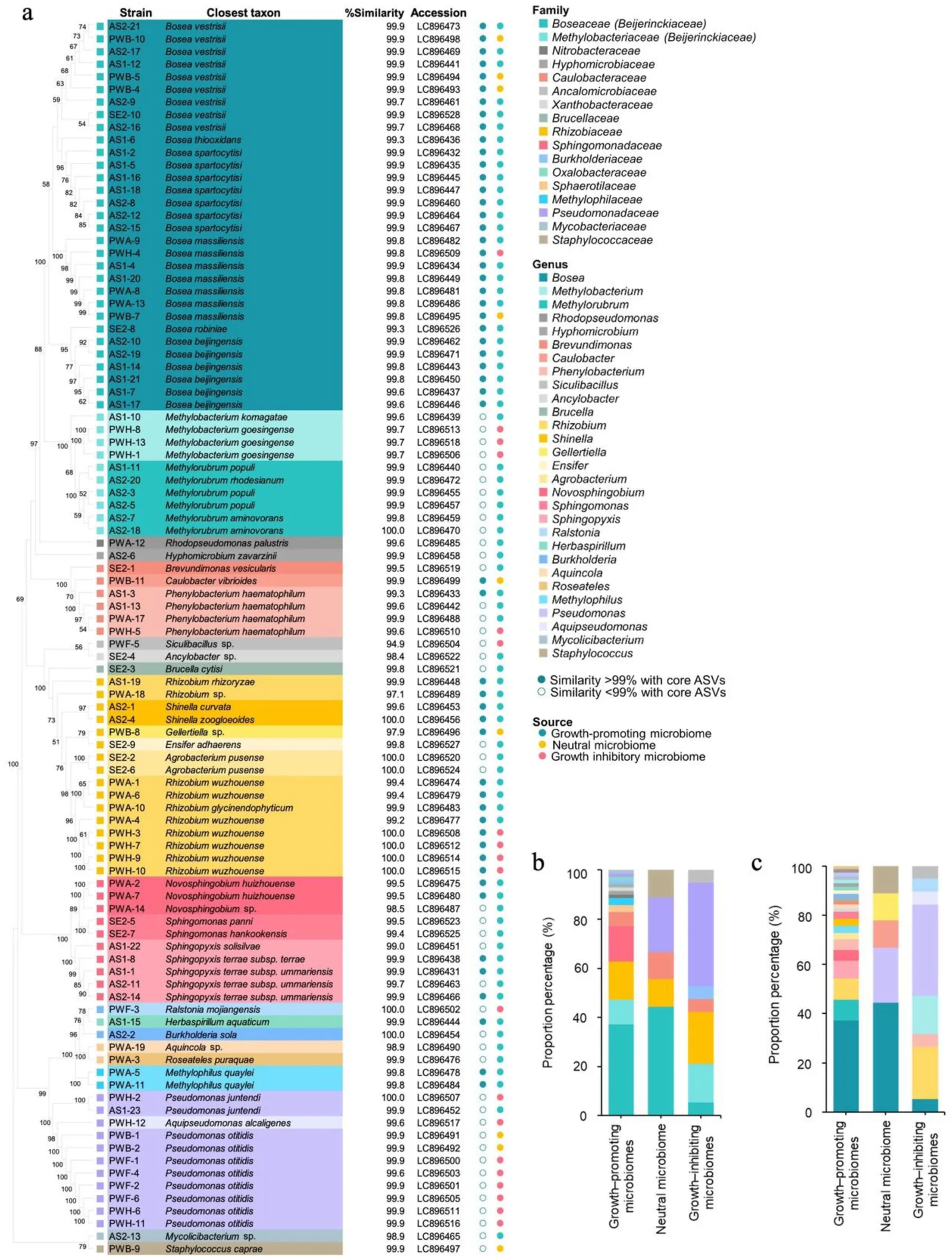
Taxonomic identification and phylogenetic relationships of 98 duckweed-associated bacteria strains isolated from 7 Wolffia globosa microbiomes. a) Phylogenetic reconstruction based on 16S rRNA gene sequences using the neighbor-joining method, showing sequence similarities to the closest taxon and correspondence to core amplicon sequence variants (ASVs) from the original microbiomes (Bioproject PRJDB18855). b) Family-level composition classified by three microbiome phenotypes. c) Genus-level composition classified based on three microbiome phenotypes.

Among these 98 isolated DAB, 54 shared > 99% 16S rRNA gene sequence similarity with 22 previously identified core ASVs at the genus level in *W. globosa* microbiomes [10]. These matched strains represented 10 genera across *Boseaceae*, *Rhizobiaceae*, *Caulobacteraceae*, *Sphingomonadaceae*, *Oxalobacteraceae*, and *Methylophilaceae*. Collectively, these results demonstrate the successful isolation of DAB representing core *W. globosa* taxa (Figure 1), evidencing the successful colonization of surrounding bacterial communities on *Wolffia*. This culture collection enabled the characterization of individual strain-level effects on *Wolffia* growth.

### 3.2. Effects of isolated DAB on Wolffia growth

#### 3.2.1. At the strain level

Of the 98 bacterial isolates, 33 (33.7%) on day 3 and 46 (46.9%) on day 6 significantly affected *W. globosa* growth (*p* < 0.05), with EPG values ranging from −28.1% to +27.8% and −14.6% to +12.9%, respectively (Figure S2 and Table S2). The remaining strains had no significant effect relative to the control or fell within a neutral range (between −5% and +5%). Among the functional strains, the number of growth-promoting strains increased from 14 on days 3 to 37 on days 6, while growth-inhibiting strains decreased from 19 to 9. This indicates that, after 6 days of cultivation, more strains exhibited significantly positive effects, whereas fewer strains showed significant negative effects after 6 days of cultivation. It should be noted that these growth effects were evaluated based on frond area, which does not fully capture all aspects of plant growth and physiological status. Therefore, in future studies, researchers should include additional parameters, such as frond number, fresh weight, dry weight, chlorophyll content, and other physiological indicators, to provide a more comprehensive evaluation.

Because the effects of DAB changed over time, only strains showing consistent promotion or inhibition at both time points (3 and 6 days) were selected as PGPB and PGIB candidates, respectively. This yielded a traceable collection of 10 PGPB and 9 PGIB strains, with the final EPG on day 6 ranging from +6.0% to +12.9% among the PGPB and from −14.6% to −6.7% among the PGIB (Figure 2). The 10 PGPB candidates comprised *Agrobacterium pusense* (SE2-2), *Bosea beijingensis* (AS1-7, AS1-14, and AS1-17), *B. spartocytisi* (AS1-5 and AS1-18), *Brucella cytisi* (SE2-3), *Herbaspirillum aquaticum* (AS1-15), *Methylorubrum populi* (AS2-5), and *Shinella curvata* (AS2-1) (Table S3). The 9 PGIB candidates comprised *Brevundimonas vesicularis* (SE2-1), *Burkholderia sola* (AS2-2), *Methylobacterium goesingense* (PWH-8), *Methylophilus quaylei* (PWA-5, PWA-11), *Novosphingobium huizhouense* (PWA-2, PWA-7), *Phenylobacterium haematophilum* (PWA-17), and *Ralstonia mojiangensis* (PWF-3) (Table S3). Collectively, these findings confirm that *W. globosa* hosts PGPB and PGIB, similar to rooted duckweed [24]. Notably, strong and consistent effects on host plant growth were observed only in a small subset of DAB (19 of 98), with most strains displaying weak or context-dependent influences, highlighting the complex, strain-specific host-microbe dynamics.

Of the 19 candidates, seven PGPB and four PGIB matched the core ASVs belonging to the genera *Bosea*, *Herbaspirillum*, *Methylophilus*, *Novosphingobium*, and *Shinella*. The PGPB matches included *B. beijingensis* (strains AS1-7, AS1-14, and AS1-17), *B. spartocytisi* (AS1-5 and AS1-18), *S. curvata* (AS2-1), and *H. aquaticum* (AS1-15). The PGIB matches were *Me. quaylei* (PWA-5 and PWA-11) and *N. huizhouense* (PWA-7 and PWA-2) (Figure 2 and Table S3). These core ASV-matched strains would be ecological important in the microbiome and potentially serve as key functional determinants of *W. globosa* growth promotion or inhibition, which are hereinafter proposed as core PGPB and core PGIB, respectively. Core microorganisms are generally considered more effectively adapted to the unique ecological challenges or contexts faced by the host species than non-core members. Studies on terrestrial plants have demonstrated that growth-promoting core strains outperform non-core strains in terms of reproducibility, functional transmission, and competitive advantage over indigenous bacteria [27],[30],[31]. Previously reported PGPB inoculants have faced challenges in establishing strong associations with duckweed because of their microbiome member-selection behavior [32],[33]. Therefore, the PGPB and PGIB strains identified in this study likely represent promising, traceable strains for microbiome engineering to sustainably enhance productivity by harnessing core PGPB as growth promoters and leveraging core PGIB as targets to develop strategies to alleviate growth inhibition, prioritizing core PGPB/PGIB. However, further studies are required to investigate their ecological roles in the microbiome.

**Figure 2.**
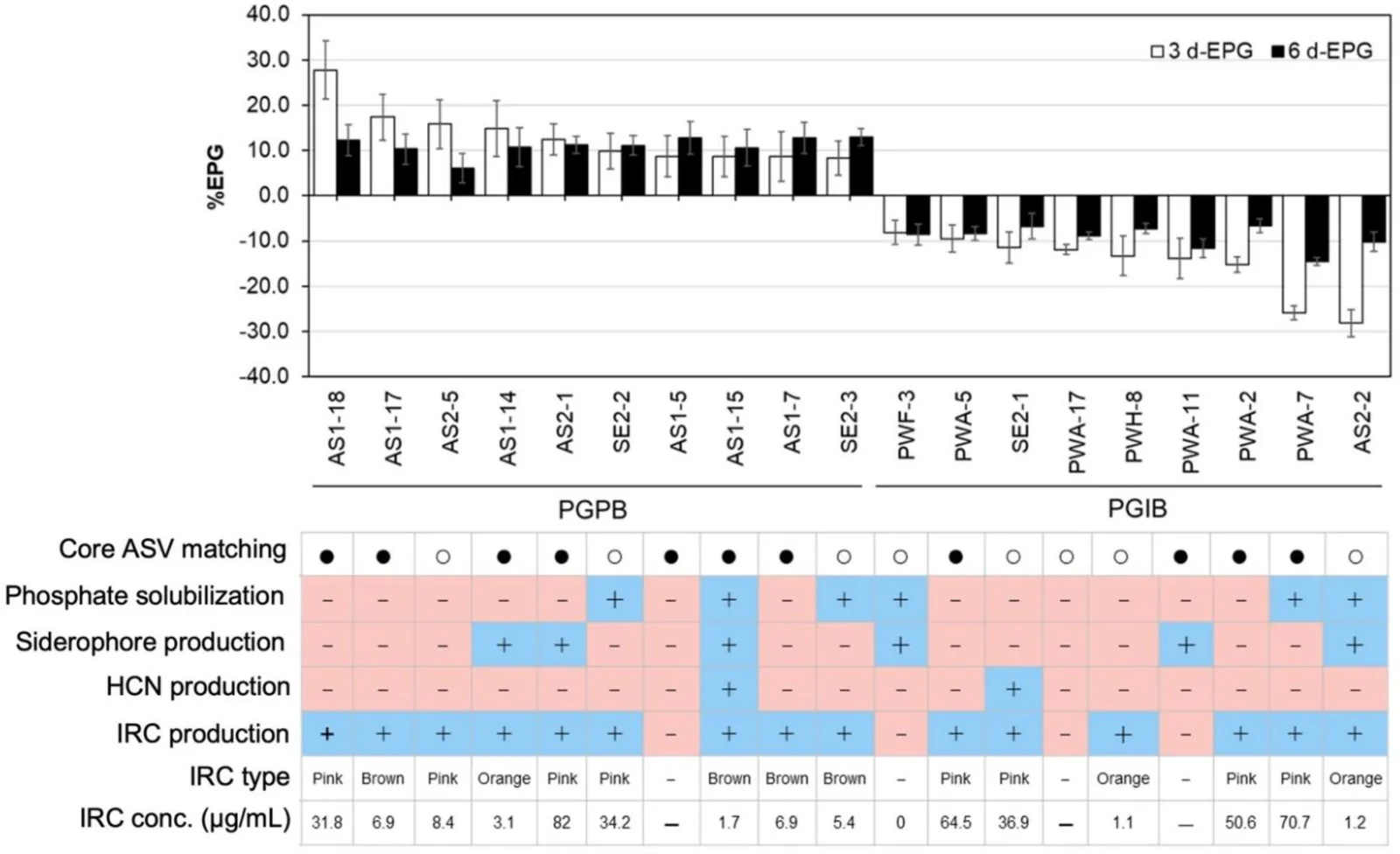
Effect on plant growth of plant growth-promoting bacteria (PGPB) and plant growth-inhibiting bacteria (PGIB) at day 3 and day 6, and their correspondence to core amplicon sequence variants (ASVs) from the original microbiomes (Bioproject PRJDB18855) [10] and physiological characteristics. “+” and “−” indicate positive and negative results for each physiological characteristic, respectively.

To the best of our knowledge, we are the first to report *Bosea* spp. as a functional PGPB in *Wolffia*. *Bosea* has been identified as a core genus within the *W. globosa* microbiome [10] and was predominantly distributed in growth-promoting microbiome phenotypes in this study, reinforcing its role as a core growth-associated genus. Furthermore, as no evidence of human pathogenicity has been documented to date [34],[35], *Bosea* spp. may be suitable candidates for application in natural environments. However, additional research encompassing genome-based functional profiling, pathogenicity assays, and assessment of their roles in plant interactions is required to confirm their functional mechanisms, biosafety, and ecological roles before practical application. In addition, *S. curvata* served as a potential PGPB persistent in *W. globosa* in this study and has been reported as a PGPB for rooted duckweed *Spirodela polyrhiza* [29], suggesting broader efficacy across rooted and rootless duckweed species. In contrast, *Novosphingobium* sp. and *Brevundimonas* sp. have been identified as PGPB in rooted duckweed [29],[36]; however, they were identified as PGIB of *W. globosa* in this study, suggesting that their functional roles may be affected by host morphology or host-secreted metabolites. In addition, *Methylophilus* was reported as a PGIB in duckweed for the first time.

#### 3.2.2. At the community level

To contextualize community-level phenotypes, we summarized the effects of isolated DAB based on their original microbiomes [10]. Strains isolated from growth-promoting microbiomes (PW_A, SE_2, AS_1, and AS_2) exhibited effects ranging from strongly inhibitory (−14.6%) to strongly promotive (+12.9%) on *Wolffia* growth after 6 days (Figure S2a). Most strains from SE_2, AS_1, and AS_2 exhibited enhanced host growth and yielded all PGPB candidates identified in this study. However, these communities also contained growth-inhibitory strains such as AS2-2, which consistently suppressed *Wolffia* growth over 6 days (Figure S2a and Table S2). This indicates that growth-promoting community phenotypes may not arise from uniformly beneficial members. Notably, although PW_A showed a slight growth-promoting effect at the community level (+5.3%) [10], none of the DAB isolated from PW_A promoted growth in monoculture (Figure S2a; Figure S3). Plausible explanations for the contradiction between strain- and community-level outcomes are as follows: (i) Functional growth promoters were uncultured or not recovered under our isolation conditions (Figure S1a and Figure S1b), and (ii) growth-promoting activity is an emergent property arising from microbial interactions (e.g., cross-feeding or cooperative metabolite production) that are not captured in monoculture assays [17].

In microbiome PW_B, which exhibited a neutral effect, most of the nine isolated strains did not significantly influence *Wolffia* growth (Figure S2b, Figure S3). Although the effects of PWB-8 and PWB-9 were statistically significant (*p* < 0.05), they remained within the neutral effect threshold (−5 to +5%). These findings suggest that this microbiome exerts a neutral influence on host growth owing to the negligible effects of individual community constituents.

Among the growth-inhibitory microbiomes (PW_F and PW_H), strains from PW_H generally suppressed *W. globosa* growth after 3 days (*p* < 0.05); however, their inhibitory effects were mitigated after 6 days (Figure S2c and Figure S3). Notably, PWH-12 initially showed a strong inhibitory effect (−24.8%), which shifted to neutral (−1.3%) after 6 days (Figure S2c and Table S2). This was probably due to the change in conditions during co-cultivation from suspension in the first stage (day 0–3) to attachment conditions in the later stage (day 3–6), which likely reduced the bacterial cell number and may account for the observed growth recovery. In contrast, among the six strains isolated from PW_F, most strains tended to slightly promote host growth, whereas only PWF-3 consistently exhibited inhibitory effects (Figure S2c and Figure S3). This may be attributed to the low number of isolates from PW_F.

Overall, the effects of individual DAB on *W. globosa* growth were diverse and dynamic, differing not only among microbiomes but also among the same taxa within the same community. These results confirm that individual communities can contain beneficial and detrimental members [24],[29], and community-level outcomes cannot be fully explained solely by culturable DAB, as discussed above regarding the PW_A contradiction. Culture-based approaches capture only a portion of the functional diversity within microbiomes (Figure S1). Despite these limitations, growth-promoting microbiomes derived from wastewater sources yielded multiple PGPB candidates, which could potentially serve as effective inocula for establishing a superior plant microbiome or for exploring PGPB [10]. Whether community-level outcomes reflect additive strain effects, emergent interactions, or the contribution of uncultured members remains an open question that warrants investigation through reconstituted synthetic community experiments [17],[32].

In addition to community-level phenotypes, the source of inoculum appears to be relevant. Microbiomes and culturable DAB derived from pond water tended to yield fewer growth-promoting strains, with most strains being neutral or inhibitory (representing the primary source of PGIB identified in this study). This may reflect the limited functional compatibility of pond-derived bacteria to the unique morphology of *Wolffia*, given the absence of this plant at the sampling sites of pond water in our other study [10].

#### 3.2.3. At higher taxonomic levels

In addition to evaluating the effects of the isolated DAB on *Wolffia* growth at the individual strain and community levels, the effects of the isolated DAB were analyzed according to taxonomic group to better understand the relationship between bacterial taxa and *Wolffia* growth.

In this analysis, taxa with mean EPG > +5% were classified as promotive; < −5% as inhibitory effects on *W. globosa* growth. Of the 17 families, three, *Brucellaceae*, *Oxalobacteraceae*, and *Boseaceae*, exhibited promotive effects, whereas two, *Burkholderiaceae* and *Methylophilaceae*, showed uniformly inhibited host growth (Figure 3a). The remaining 12 families fell within the neutral range, whose members did not uniformly affect host growth and displayed variable outcomes ranging from inhibition to promotion (Figure 3a and Figure S4a). The promotive effect observed in *Boseaceae* and inhibitory effects in *Burkholderiaceae* are consistent with our other findings based on 16S rRNA amplicon sequencing [10], which showed positive correlations with *Beijerinckiaceae* and negative correlations with *Burkholderiaceae* [10], supporting the consistency between culture-independent and culture-based study.

At the genus level, among the 29 genera, promotive effects were distributed across six genera, *Brucella*, *Herbaspirillum*, *Shinella*, *Agrobacterium*, *Sphingopyxis*, and *Bosea*, with no inhibiting strains identified among them (Figure 3b). Notably, within *Bosea*, the most extensively isolated genus in this study, more than half of the strains (17/31) promoted host growth (Figure S4a). Conversely, inhibitory effects were observed in five genera, *Burkholderia*, *Methylophilus*, *Ralstonia*, *Brevundimonas*, and *Novosphingobium*, where all strains uniformly inhibited host growth and most *Novosphingobium* strains (2/3) also exhibiting inhibitory effects (Figure 3b and Figure S4b), suggesting that they may serve as indicators of growth-inhibiting potential.

This analysis revealed that the host growth effects of DAB were more similar among members within the same genus than among members classified only at the family level. For example, within the family *Sphingomonadaceae*, *Sphingopyxis* exhibited promotive effects, whereas *Novosphingobium* exhibited inhibitory effects. Overall, these findings suggest that promotive and inhibitory effects were associated with bacterial taxonomy, but they were also strongly strain-dependent, as evidenced by the variability observed among strains within the same genus (Figure 3b and Figure S4b), consistent with patterns reported in terrestrial plants [37]. This pattern is similar to the hierarchical predictive pattern in which family provides broad classification, genus offers intermediate prediction, and strain represents the most informative level for predicting host growth effects. However, these observations were derived from a single experimental system with uneven numbers of culturable DAB across taxonomic groups. Broader validation is required to confirm the generality of this pattern.

**Figure 3.**
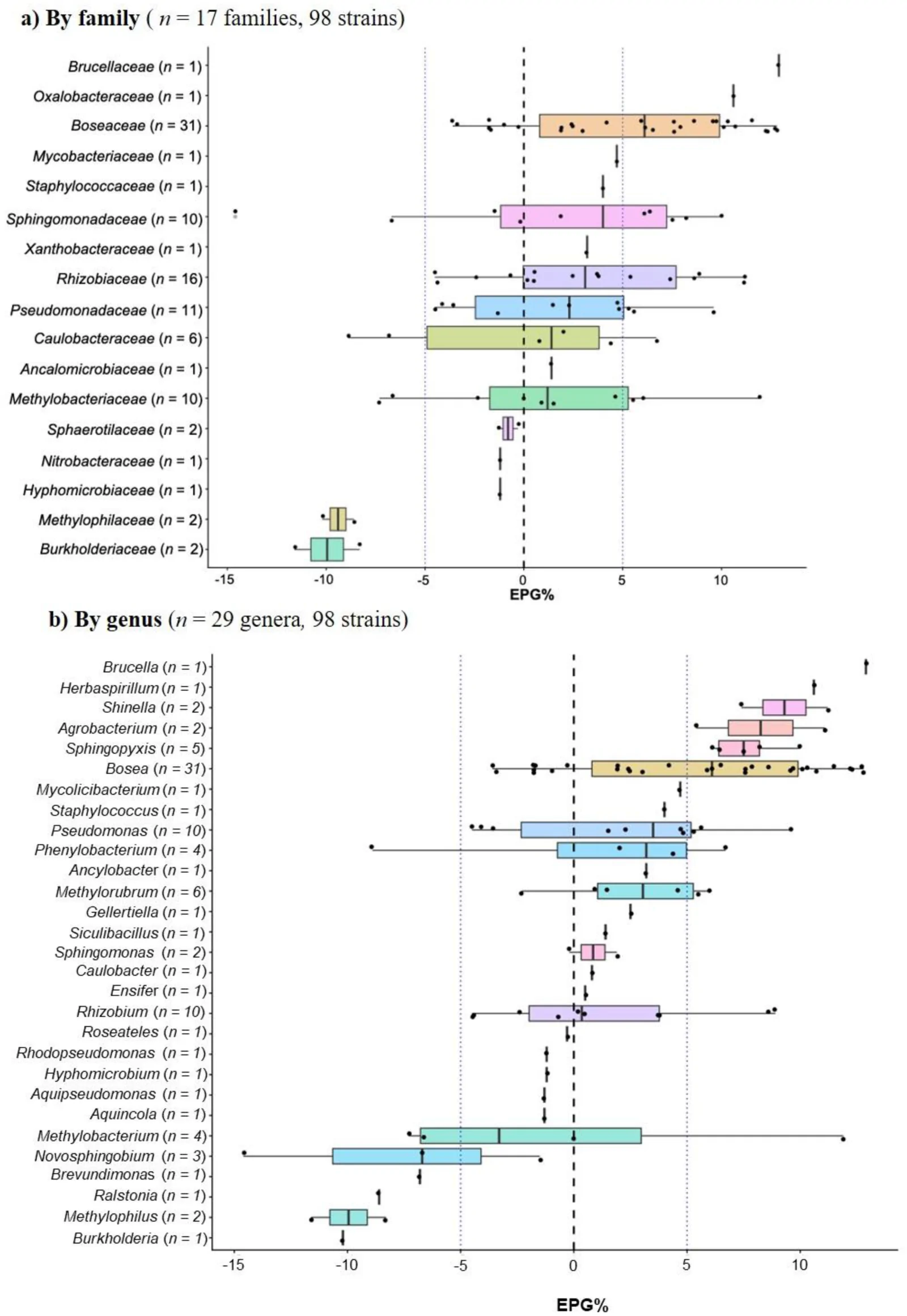
Box-and-whisker plots showing distribution of effect on plant growth of 98 DAB strains after 6 days of cultivation at higher taxonomic levels: a) family level and b) genus level. The black dashed line indicates growth relative to that of the control. The blue dashed line marks the EPG threshold for a neutral effect.

### 3.3. Physiological characterization of DAB affecting Wolffia growth

All isolated DAB were screened for four general physiological traits associated with plant growth promotion. Of the 98 strains, 88 (89.8%) were positive for at least one trait, with only one strain positive for all four traits (Table S2). Phosphate solubilization, siderophore, and HCN production were detected in 24 (24.4%), 26 (26.5%), and 6 (6.1%) strains, respectively. IRC production was the most common (85 strains, 86.7%), with estimated concentrations ranging from 0.1 to 175.4 µg/mL. Based on colorimetric detection and wavelength scanning, the IRCs were broadly classified into three types: Pink (48 strains), orange (26 strains), and brown (11 strains). Although precise identification required liquid chromatography-mass spectrometry analysis, the pink color change was suspected to be due to indole-3-acetic acid (IAA), indole-acetamide (IAM), or indole-3-pyruvic acid (IPA); orange was likely to be due to indole-3-butyric acid (IBA); and brown was thought to be due to indole [24].

Based on multiple linear regression assessing the relationship between EPG and physiological traits, only IRC production was significantly associated with increased *Wolffia* growth (β = 3.776, *p* = 0.043; Table 1), whereas the other traits showed no statistically significant association. This indicates that DAB strains capable of IRC production induce significantly greater host growth than the strains without IRC production. Although the IRC production showed a significant positive association, the overall model did not reach statistical significance (R^2^ = 0.055, adjusted R^2^ = 0.014, *p* = 0.257), indicating that these four traits collectively explained only a small proportion of the variance in host growth.

**Table 1.** Association of physiological traits of duckweed-associated bacteria with their effects on *Wolffia globosa* growth using multiple linear regression.

Factor	Coefficient (β)	SE	t value	*p*-value
Phosphate solubilization	−0.508	1.454	−0.349	0.728
Siderophore production	0.650	1.421	0.457	0.648
Hydrogen cyanide production	1.817	2.595	0.700	0.486
IRC production	3.776	1.841	2.051	0.043
R^2^ = 0.05494, Adjusted R^2^ = 0.01429, *p*-value: 0.2569			
IRC type				
Pink-type	3.492	1.860	1.877	0.063
Orange type	3.223	2.020	1.595	0.114
Brown type	7.550	2.437	3.098	0.002
R^2^ = 0.09295, Adjusted R^2^ = 0.064, *p*-value: 0.0265			

IRC, indole related-compounds.

To further determine which IRC type contributes to the host growth promotion, based on the different color changes observed in the Salkowski assay among the IRC-producing strains, a second model was performed. Only brown type IRC production was significantly associated with increased host growth relative to non-producers (β = 7.550, *p* = 0.002; Table 1), highlighting the specialized role of brown-type IRCs in the interaction between *Wolffia* and its DAB. Pink-type IRC production showed a positive but non-significant trend (β = 3.492, *p* = 0.063), and orange-type IRC production showed no significant association (β = 3.223, *p* = 0.114). This model explained a greater proportion of variance in host growth than the full four-trait model (R^2^ = 0.093, adjusted R^2^ = 0.064, *p* = 0.027).

IRCs are involved in plant phytohormones, which regulate plant growth and development and play significant roles in plant-microbe interactions in terrestrial environments, with IAA being a major naturally occurring auxin [38]. While a large majority of our DAB collection could synthesize IRCs (86.7%), the lack of significant association between *Wolffia* growth and pink-type (likely IAA/IAM/IPA) or orange-type (likely IBA) suggests that the classic IAA-mediated growth pathways known in terrestrial plants may not be the primary driver of *Wolffia* growth promotion. This aligns with other findings demonstrating a duckweed genus-specific correlation, where brown-type IRC production is likely associated with DAB derived from *Wolffia*, whereas pink-type IRC production is more typical for *Lemna* DAB [25]. Collectively, it was suggested that manipulation of IRC pathways, particularly indole signaling pathways, likely promotes *W. globosa* growth.

Given the rootless morphology of *Wolffia*, this plant may be less responsive to IAA-producing bacteria than rooted duckweeds, which typically promote growth by modulating root architecture, such as shortening primary roots and increasing root hair density [39],[40]. Instead, indole may function as a specialized signaling molecule within the *Wolffia* holobiont, as it is known to be involved in bacterial biofilm formation and quorum sensing [41],[42], and may facilitate effective colonization of the *Wolffia* fronds. This is likely supported by the evidence that brown-type IRC production was exclusively found in our PGPB strains (strains SE2-3, AS1-15, and AS1-17 in Figure 2), all of which exhibited biofilm-forming capabilities (data not shown). Gilbert et al. [40] demonstrated that indole-producing DAB isolated from *Wolffia* can colonize the leaf surface. This supports the hypothesis that indole facilitates more effective establishment and colonization of *Wolffia* fronds, which may be a prerequisite for PGPB to promote host growth in a rootless duckweed.

However, the functional roles of specific IRCs in *Wolffia* growth require further validation. Although the Salkowski colorimetric assay is useful for screening, it cannot precisely distinguish IRC derivatives. For example, we observed pink-type IRCs in the PGPB and PGIB collections. Gilbert et al. [25] confirmed using liquid chromatography mass spectrometry analysis that pink-type-producing DAB primarily produce IAA alongside low signals of the other IRCs. Therefore, the role of these IRCs in *W. globosa* growth could not be completely understood in this study and remains to be fully elucidated.

The role of general physiological traits in duckweed growth remains contentious. Ishizawa et al. [23] found that bacterial phosphate solubilization significantly correlates with *L. minor*, while IAA production shows no correlation. Another study revealed that the external application of IAA [0–50 µM (0–8.76 µg/mL)] does not promote growth in *L. minor* [43]. The absence of a significant association between *Wolffia* growth and general physiological traits other than IRC production in this study further emphasizes the unique importance of IRCs in this aquatic plant-microbe interaction. However, this does not exclude the possible contribution of other bacterial functions. For example, nitrogen fixation and 1-aminocyclopropane-1-carboxylate deaminase activity, which were not examined in this study, may also contribute to host growth promotion and improve the prediction of host growth responses. Nevertheless, the fact that most of our traceable PGPB strains possessed multiple physiological traits (Figure 2) suggests that growth promotion may not occur via a single specific mechanism, but rather as a synergistic result of multiple physiological traits within a PGPB strain.

## 4. Conclusions

This study provides a culture-based characterization of DAB isolated from *W. globosa* microbiomes, revealing diverse effects on host growth, ranging from promotion to inhibition, which are strain-specific, taxonomically diverse, and dynamic over time. Of the 98 isolated DAB, 19 strains consistently affected *W. globosa* growth throughout the experiment, yielding 10 PGPB and 9 PGIB candidates. Importantly, seven PGPB and four PGIB matched core ASVs of the *W. globosa* microbiome and were proposed as core PGPB and core PGIB, representing ecologically established DAB serving as potential functional determinants of host growth. Growth-promoting microbiomes derived from wastewater sources were likely enriched with functional-promoting strains for *W. globosa* growth. Taxonomic analysis further revealed that promotive and inhibitory phenotypes occurred across six and five genera, respectively, with effects strongly determined at the strain level. The production of IRC, more specifically indole, has emerged as a potential physiological determinant of host growth, suggesting its specialized role in this rootless duckweed. These findings provide a traceable strain collection for the engineering of the *Wolffia* holobiont and offer practical insights into microbiome control strategies to sustainably enhance productivity, including harnessing core PGPB as growth promoters and targeting core PGIB for suppression through predator/phage-based biocontrol methods.

## Use of AI tools declaration

The authors declare they have not used Artificial Intelligence (AI) tools in the creation of this article.


