# Supplementary material for: Characterization of duckweed-associated bacteria from the Wolffia globosa microbiome: Strain-level effects on host growth and key determinants
Source: AIMS Microbiol. 2026 Jul 30;12(3):508–25. doi: 10.3934/microbiol.2026022 (PMC13652746; doi:10.3934/microbiol.2026022)
Supplement: Supplementary file 1 [file microbiol-12-03-022-s001.pdf]

Microbiology

AIMS Microbiology, 12(3): 508–525.
DOI: 10.3934/microbiol.2026022
Received: 08 May 2026
Revised: 06 July 2026
Accepted: 17 July 2026
Published: 30 July 2026

https://www.aimspress.com/journal/microbiology

Research article

Characterization of duckweed-associated bacteria from the *Wolffia globosa* microbiome: Strain-level effects on host growth and key determinants

Yuparat Saimee, Ryusuke Okada, Daisuke Inoue* and Michihiko Ike*

Division of Sustainable Energy and Environmental Engineering, The University of Osaka, 2-1 Yamadaoka, Suita, Osaka 565-0871, Japan

* **Correspondence:** Email: d.inoue@see.eng.osaka-u.ac.jp; ike@see.eng.osaka-u.ac.jp; Tel: +81668797673; Tel: +81668797672.

Supplementary

Supplementary Text 1 Image processing procedure based on RGB-to-HSV color conversion using Python on Google Colab (https://colab.research.google.com).

```
#Environmental setup and library imports
%matplotlib inline
import cv2
import numpy as np
from google.colab.patches import cv2_imshow
from google.colab import files
#Uploading multiple image file
uploaded_files = files.upload()
# Setting numeric display precision
np.set_printoptions(precision=10)
# Initialize a list to store the results
results = []
# Looping through each uploaded file
for filename, content in uploaded_files.items():
# Read the image from the content
    img = cv2.imdecode(np.frombuffer(content, dtype=np.uint8), -1)
# Converting RGB to HSV for green color representing Wolffia frond
hsv = cv2.cvtColor(img, cv2.COLOR_BGR2HSV)
#Definging the green color range and creating a binary mask
hsv_min = np.array([30, 100, 0])
hsv_max = np.array([90, 255, 255])
mask = cv2.inRange(hsv, hsv_min, hsv_max)
#Applying the mask to the original image
masked_img = cv2.bitwise_and(img, img, mask=mask)
#Quantifying green coverage
green_pixels = np.count_nonzero(mask == 255)   # detected green pixels
total_pixels = mask.size                        # total pixels in image
coverage_pct = green_pixels / total_pixels * 100
# Display the mask area for visual confirmation
print(f"\n{filename}")
print(f"   Green pixels detected : {green_pixels}")
print(f"   Total pixels          : {total_pixels}")
print(f"   Coverage              : {coverage_pct:.4f}%")
cv2_imshow(masked_img)
#Storing results
results.append([filename, green_pixels, total_pixels, round(coverage_pct, 6)])
#Exporting results to CSV
csv_filename = "wolffia_results.csv"
with open(csv_filename, "w") as csv_file:
```

```
    csv_file.write("Filename,Green_Pixels,Total_Pixels,Coverage_Pct\n")
    for result in results:
        csv_file.write(f"{result[0]},{result[1]},{result[2]},{result[3]}\n")
#Downloading the CSV
print(f"\n Results saved to {csv_filename}")
files.download(csv_filename)
```

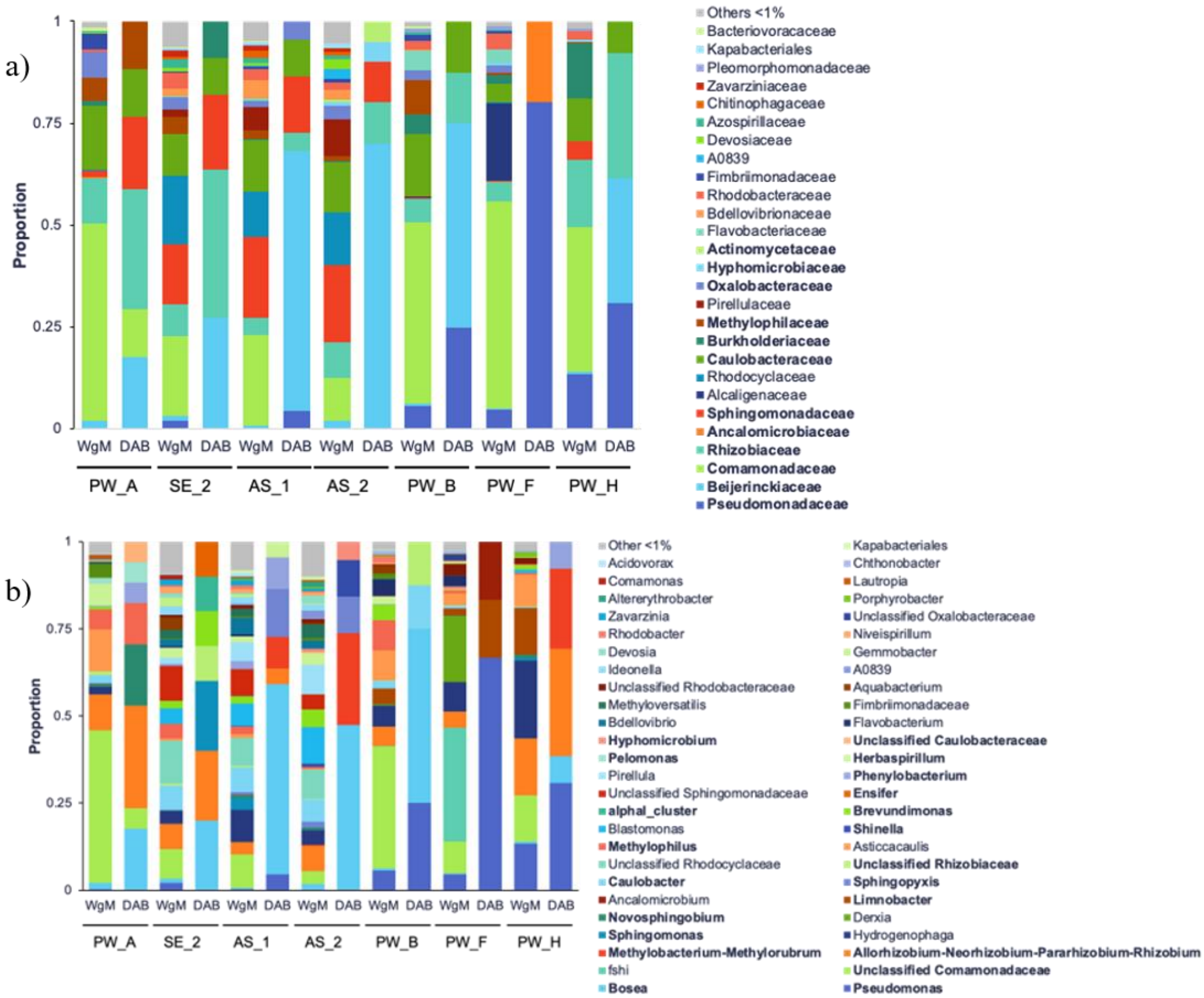

Figure S1. Comparison of bacterial taxa composition between *Wolffia globosa* microbiomes (WgM) and isolated duckweed-associated bacteria (DAB); a) Family level and b) genus level. Stacked bar plots represent the relative abundance of bacterial taxa in each microbiome sample and the proportional distribution of isolated DAB strains obtained from the same microbiome. Taxa with relative abundance < 1% are grouped as "Other < 1%." Names in bolded black font are taxa from which DABs were isolated and tested on *W. globosa* in the experiment.

Table S1. Number of strains obtained from each microbiome.

Communities	Number of strains	Community level effect*	Community diversity (Shannon index)*	Sources of the derived Wolffia microbiome
PW_A	17	Promotion	3.0	Freshwater pond
SE_2	10	Promotion	4.0	Activated sludge
AS_1	22	Promotion	4.2	Activated sludge
AS_2	21	Promotion	4.2	Activated sludge
PW_B	9	Neutrality	3.0	Freshwater pond
PW_F	6	Inhibition	2.6	Freshwater pond
PW_H	13	Inhibition	2.7	Freshwater pond

*The effect of the overall microbiome on *W. globosa* growth derived from Saimee et al., 2025.

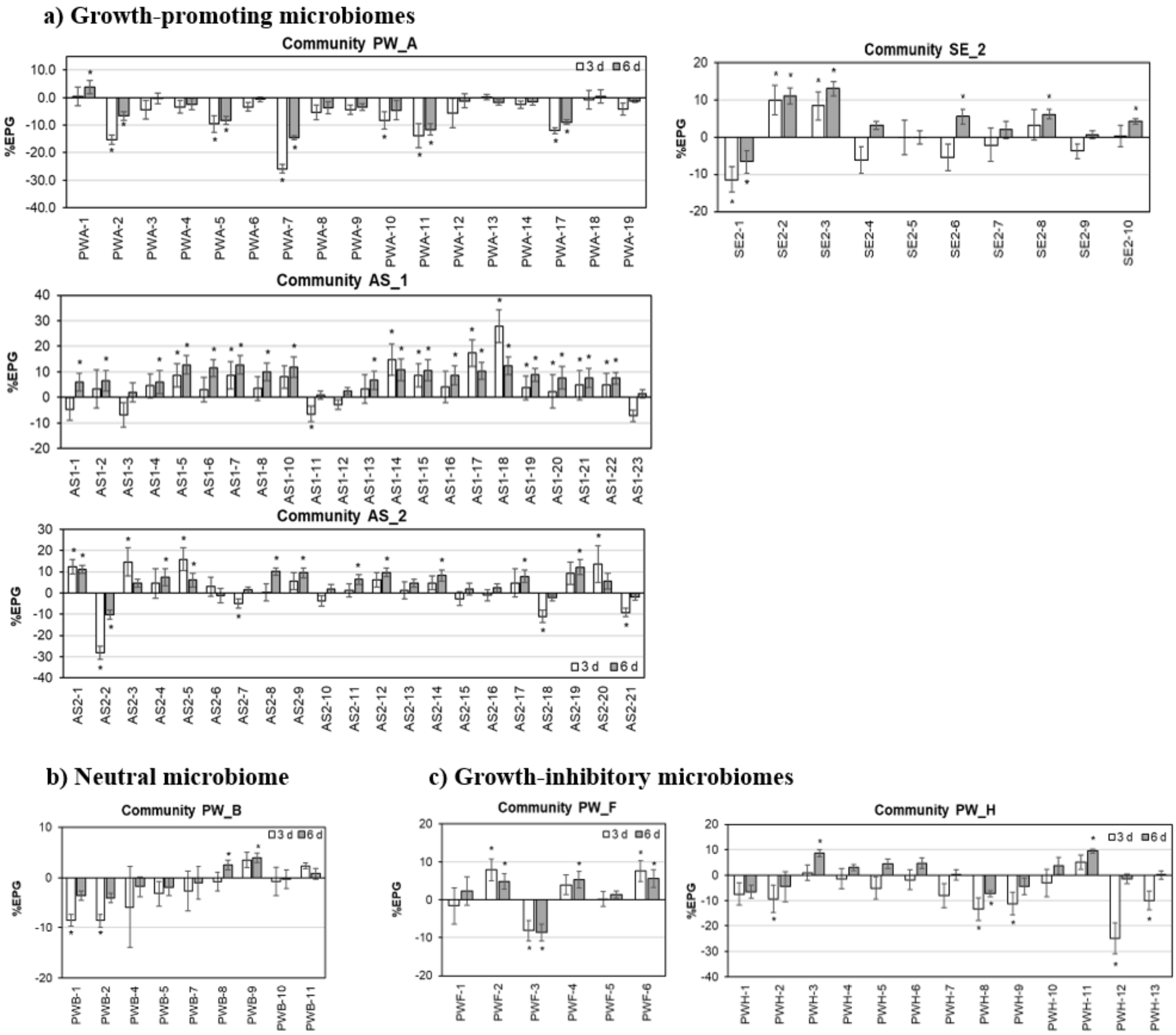

Figure S2. Effect on plant growth (EPG) of isolated strains after 3 and 6 days. a) Strains derived from growth-promoting microbiomes, b) strains from a neutral microbiome, and c) strains from growth-inhibitory microbiomes. Error bars represent standard deviations (n = 5). Asterisks indicate significant differences (one-way ANOVA, Tukey post hoc test; $p < 0.05$)

Table S2. Effect on plant growth (%EPG) of 98 DAB strains compared to the control after 3 d and 6 d of cultivation, and physiological characteristics of each strain. EPG values shown in the table are mean ± SD (n = 5). Letters indicate significant differences within the community ($p < 0.05$, Tukey's HSD test). Asterisks (*) denote significant differences ($p < 0.05$, Tukey's HSD test) in the change of EPG between 3 d and 6 d of the strain. P sol., phosphate solubilization; Sid., siderophore production; HCN, hydroden cyanide production; IRCs conc., indole-related compounds concentration (μg/mL); Type of IRCs., type of indole-related compounds detected by wavelength scanning. "+" and "−" indicate positive and negative results for each physiological characteristic, respectively.

Strains	3d EPG (%)		6 d EPG (%)		3d vs 6d	P sol.	Sid.	HCN	IRCs conc.	Type of IRCs.
Growth-promoting microbiome										
Community PW_A										
PWA-1	0.4 ± 3.5	a	3.8 ± 2.3	a	*	+	−	−	13.6	Orange
PWA-2	−15.2 ± 1.7	f	−6.7 ± 1.5	de	*	−	−	−	50.6	Pink
PWA-3	−4.4 ± 3.4	abc	−0.3 ± 1.9	abc	*	−	−	−	64	Pink
PWA-4	−3.3 ± 2.3	abc	−2.4 ± 2.1	bcd	ns	−	−	−	62	Pink
PWA-5	−9.5 ± 3.0	cdef	−8.3 ± 1.5	ef	ns	−	−	−	64.5	Pink
PWA-6	−3.4 ± 1.6	abc	−0.7 ± 0.9	abc	*	+	−	−	59.8	Pink
PWA-7	−25.9 ± 1.6	g	−14.6 ± 0.8	g	*	+	−	−	70.7	Pink
PWA-8	−5.3 ± 2.7	abcd	−3.6 ± 2.1	bcd	*	−	−	−	−	No color change
PWA-9	−4.4 ± 1.7	abc	−3.4 ± 1.2	bcd	ns	−	−	−	77.2	Pink
PWA-10	−8.4 ± 3.1	bcde	−4.5 ± 3.5	cde	*	+	−	−	51.1	Pink
PWA-11	−13.9 ± 4.4	ef	−11.6 ± 2.1	fg	ns	−	+	−	−	No color change
PWA-12	−5.6 ± 5.3	abcd	−1.2 ± 2.6	bc	ns	−	−	−	9.9	Orange
PWA-13	0.1 ± 1.0	a	−1.7 ± 0.9	bc	*	−	−	−	7.6	Orange
PWA-14	−2.6 ± 1.3	ab	−1.5 ± 1.3	bc	ns	−	−	−	63.4	Pink
PWA-17	−11.9 ± 1.1	def	−8.9 ± 0.8	ef	*	−	−	−	−	No color change
PWA-18	−0.7 ± 3.4	a	0.5 ± 2.4	ab	ns	+	−	−	14.3	Orange
PWA-19	−4.2 ± 2.2	abc	−1.3 ± 0.5	bc	*	+	−	−	5.8	Orange
Community SE_2										
SE2-1	−11.5 ± 3.5	f	−6.8 ± 2.9	f	*	−	−	+	36.9	Pink
SE2-2	9.8 ± 4.0	a	11.1 ± 2.2	a	ns	+	−	−	34.2	Pink
SE2-3	8.3 ± 3.8	ab	12.9 ± 1.9	a	ns	+	−	−	5.4	Brown
SE2-4	−6.2 ± 3.7	ef	3.2 ± 1.2	bcde	*	−	−	+	14.2	Pink
SE2-5	−0.3 ± 4.6	cde	−0.2 ± 1.7	e	ns	−	−	−	37.1	Pink
SE2-6	−5.6 ± 3.5	def	5.4 ± 2.0	bc	*	+	−	−	48.2	Pink
SE2-7	−2.2 ± 4.5	cde	1.9 ± 2.3	cde	*	−	−	−	33.8	Pink
SE2-8	3.2 ± 4.0	bc	6.1 ± 1.3	b	ns	−	−	−	22.7	Pink
SE2-9	−3.8 ± 2.0	de	0.5 ± 1.0	de	*	−	−	−	6.3	Orange
SE2-10	0.2 ± 2.9	cd	4.2 ± 0.8	bcd	*	−	−	−	−	No color change

Continued on next page

Strains	3d EPG (%)		6 d EPG (%)		3d vs 6d	P sol.	Sid.	HCN	IRCs conc.	Type of IRCs.
Community AS_1										
AS1-1	−4.7 ± 4.4	fgh	6.1 ± 3.5	defg	*	−	−	−	8.6	Brown
AS1-2	3.3 ± 7.5	def	6.5 ± 4.1	cdefg	ns	−	−	−	17.3	Pink
AS1-3	−6.8 ± 4.9	h	2.0 ± 3.8	efg	*	−	−	−	47.4	Orange
AS1-4	4.6 ± 4.7	de	5.9 ± 4.5	defg	ns	−	−	−	4.9	Orange
AS1-5	8.7 ± 4.6	bcd	12.7 ± 3.6	a	*	−	−	−	−	No color change
AS1-6	3.1 ± 4.9	def	11.5 ± 3.3	abcd	*	−	−	−	1.1	Brown
AS1-7	8.6 ± 5.4	bcd	12.8 ± 3.5	a	*	−	+	−	0.4	Orange
AS1-8	3.5 ± 4.7	def	10.0 ± 3.6	abcd	*	+	+	−	25.1	Pink
AS1-10	8.0 ± 4.5	cd	11.9 ± 4.0	abc	ns	−	−	+	45.4	Pink
AS1-11	−6.5 ± 3.0	gh	0.9 ± 1.6	g	*	+	−	−	10	Orange
AS1-12	−2.9 ± 1.9	efgh	2.5 ± 1.4	efg	*	−	+	−	30	Pink
AS1-13	3.3 ± 5.5	def	6.7 ± 3.6	bcdef	*	−	−	−	59.2	Pink
AS1-14	14.8 ± 6.2	bc	10.7 ± 4.3	abcd	*	−	+	−	3.1	Orange
AS1-15	8.7 ± 4.4	bcd	10.6 ± 4.1	abcd	ns	+	+	+	1.7	Brown
AS1-16	4.1 ± 6.2	def	8.6 ± 3.8	abcd	ns	+	+	−	18.4	Pink
AS1-17	17.4 ± 5.1	b	10.3 ± 3.4	abcd	*	−	−	−	6.9	Brown
AS1-18	27.8 ± 6.4	a	12.3 ± 3.5	ab	*	−	−	−	31.8	Pink
AS1-19	3.8 ± 4.7	def	8.9 ± 2.5	abcd	*	+	+	−	2.2	Orange
AS1-20	2.3 ± 6.5	defg	7.6 ± 4.4	abcde	*	+	+	−	29.3	Pink
AS1-21	4.8 ± 5.7	de	7.6 ± 3.7	abcde	ns	+	−	+	47.1	Pink
AS1-22	4.8 ± 4.6	de	7.5 ± 2.2	abcde	ns	−	−	−	0.2	Orange
AS1-23	−7.2 ± 2.3	h	1.5 ± 1.7	fg	*	−	−	−	−	No color change
Community AS_2										
AS2-1	12.4 ± 3.5	abc	11.2 ± 1.9	ab	ns	−	+	−	82	Pink
AS2-2	−28.1 ± 3.0	i	−10.2 ± 2.2	j	*	+	+	−	1.2	Orange
AS2-3	14.6 ± 6.7	ab	4.6 ± 1.8	cdefgh	*	−	+	−	16.4	Pink
AS2-4	4.5 ± 6.9	bcdef	7.4 ± 3.9	abcdef	ns	−	−	−	111.1	Pink
AS2-5	15.8 ± 5.4	a	6.0 ± 3.2	bcdefg	*	−	−	−	8.4	Pink
AS2-6	2.9 ± 4.5	cdef	−1.2 ± 3.3	hi	*	−	−	−	18.9	Pink
AS2-7	−4.9 ± 2.3	fgh	1.5 ± 1.4	ghi	*	+	−	−	2.2	Orange
AS2-8	0.4 ± 4.0	defg	10.1 ± 1.7	abc	*	−	−	−	26.5	Pink
AS2-9	5.6 ± 4.0	abcde	9.6 ± 2.3	abc	*	−	−	−	21.6	Pink
AS2-10	−3.7 ± 2.4	efgh	1.9 ± 2.0	efghi	*	−	−	−	6	Brown
AS2-11	1.2 ± 3.2	defg	6.4 ± 2.3	abcdefg	*	+	−	−	25	Orange
AS2-12	6.2 ± 3.3	abcde	9.7 ± 2.0	abc	*	−	−	−	35.8	Orange
AS2-13	1.3 ± 4.1	def	4.7 ± 1.9	cdefg	ns	−	+	−	45.6	Orange
AS2-14	4.8 ± 3.2	bcdef	8.2 ± 2.7	abcd	*	−	−	−	33.3	Brown
AS2-15	−2.7 ± 3.2	efgh	1.9 ± 2.8	fghi	*	−	−	+	47.3	Brown
AS2-16	−1.1 ± 2.6	defgh	2.4 ± 1.9	defghi	*	−	−	−	0.6	Orange
AS2-17	4.6 ± 6.6	bcdef	7.9 ± 2.9	abcde	ns	−	−	−	31.4	Brown
AS2-18	−11.0 ± 3.0	h	−2.3 ± 1.5	i	*	−	−	−	45.8	Brown
AS2-19	9.3 ± 5.3	abcd	12.2 ± 3.5	a	ns	−	−	−	40.5	Orange
AS2-20	13.6 ± 8.6	ab	5.5 ± 3.7	bcdefg	*	−	−	−	38.4	Brown
AS2-21	−9.2 ± 2.0	gh	−1.8 ± 1.5	i	***	−	−	−	33.3	Orange

Continued on next page

Strains	3d EPG (%)		6 d EPG (%)		3d vs 6d	P sol.	Sid.	HCN	IRCs conc.	Type of IRCs.
Neutral microbiome										
Community PW_B										
PWB-1	−8.5 ± 1.2	b	−3.6 ± 0.8	d	*	−	+	−	71.7	Pink
PWB-2	−8.6 ± 1.3	b	−4.1 ± 1.0	d	*	−	+	−	32.5	Pink
PWB-4	−5.9 ± 8.0	b	−1.8 ± 1.9	cd	ns	−	−	−	−	No color change
PWB-5	−3.2 ± 2.5	ab	−1.8 ± 1.7	cd	ns	−	−	−	−	No color change
PWB-7	−2.7 ± 3.9	ab	−1.0 ± 3.2	bcd	*	−	−	−	24.7	Pink
PWB-8	−0.9 ± 1.9	ab	2.5 ± 1.0	ab	*	−	−	−	0.1	Orange
PWB-9	3.4 ± 1.5	a	4.0 ± 0.9	a	ns	+	−	−	12.6	Pink
PWB-10	−0.8 ± 2.8	ab	−0.3 ± 1.8	bcd	ns	−	−	−	−	No color change
PWB-11	2.3 ± 0.6	a	0.8 ± 1.1	abc	ns	−	−	−	2.3	Orange
Growth-inhibitory community										
Community PW_F										
PWF-1	−1.6 ± 4.7	c	2.3 ± 3.7	bc	*	−	+	−	71.7	Pink
PWF-2	7.9 ± 2.8	a	4.8 ± 2.1	ab	*	−	+	−	67.7	Pink
PWF-3	−8.1 ± 2.7	d	−8.6 ± 2.3	d	ns	+	+	−	−	No color change
PWF-4	4.0 ± 2.6	ab	5.3 ± 2.1	ab	ns	−	+	−	53	Pink
PWF-5	0.2 ± 1.9	bc	1.4 ± 0.9	c	ns	+	−	−	−	No color change
PWF-6	7.6 ± 2.8	a	5.6 ± 2.3	a	*	−	+	−	91.8	Pink
Community PW_H										
PWH-1	−7.4 ± 4.4	bcdef	−6.6 ± 2.6	e	ns	−	−	−	1.8	Orange
PWH-2	−9.3 ± 5.4	cdef	−4.5 ± 6.0	de	*	+	+	−	17.8	Pink
PWH-3	0.9 ± 3.0	ab	8.6 ± 1.4	ab	*	−	+	−	70	Pink
PWH-4	−1.4 ± 3.9	abc	3.0 ± 1.2	bc	*	−	−	−	−	No color change
PWH-5	−5.0 ± 4.3	bcdef	4.4 ± 1.8	abc	*	+	+	−	21.2	Pink
PWH-6	−1.8 ± 3.9	abcd	4.7 ± 2.1	abc	*	−	+	−	67.3	Pink
PWH-7	−8.1 ± 4.8	cdef	0.2 ± 2.0	cd	*	−	−	−	81.6	Pink
PWH-8	−13.3 ± 4.4	f	−7.3 ± 1.2	e	*	−	−	−	1.1	Orange
PWH-9	−11.2 ± 4.5	ef	−4.4 ± 3.3	de	*	−	−	−	67.3	Pink
PWH-10	−3.0 ± 5.4	abcde	3.7 ± 3.4	abc	*	−	−	−	–	No color change
PWH-11	5.2 ± 2.9	a	9.6 ± 0.9	a	*	−	−	−	175.4	Pink
PWH-12	−24.8 ± 6.2	g	−1.3 ± 1.9	cde	*	−	+	−	7.7	Pink
PWH-13	−10.0 ± 3.7	def	0.0 ± 1.6	cd	*	−	+	−	1.2	Orange

Table S3. List of DAB strains isolated from *W. globosa* microbiomes function as proposed PGPB and PGIB.

Strain	Closest taxa	Acession	%similarity	Core ASV	Closest ASV
PGPB					
SE2-2	*Agrobacterium pusense*	LC896520	100	○	○
SE2-3	*Brucella cytisi*	LC896521	99.77	○	○
AS1-5	*Bosea spartocytisi*	LC896435	99.85	●	ASV_0085
AS1-7	*Bosea beijingensis*	LC896437	99.56	●	ASV_0085
AS1-14	*Bosea beijingensis*	LC896443	99.77	●	ASV_0085
AS1-15	*Herbaspirillum aquaticum*	LC896444	99.86	●	ASV_0120
AS1-17	*Bosea beijingensis*	LC896446	99.62	●	ASV_0085
AS1-18	*Bosea spartocytisi*	LC896447	99.85	●	ASV_0085
AS2-1	*Shinella curvata*	LC896453	99.63	●	ASV_0183
AS2-5	*Methylorubrum populi*	LC896457	99.93	○	○
PGIB					
Strain	Closest taxa	Acession	%similarity	Core ASV	Closest ASV
PWA-2	*Novosphingobium huizhouense*	LC896475	99.48	●	ASV_0342
PWA-5	*Methylophilus quaylei*	LC896478	99.78	●	ASV_0024
PWA-7	*Novosphingobium huizhouense*	LC896480	99.48	●	ASV_0342
PWA-11	*Methylophilus quaylei*	LC896484	99.78	●	ASV_0024
PWA-17	*Phenylobacterium haematophilum*	LC896488	99.93	○	○
SE2-1	*Brevundimonas vesicularis*	LC896519	99.52	○	○
AS2-2	*Burkholderia sola*	LC896454	100	○	○
PWF-3	*Ralstonia mojiangensis*	LC896502	100	○	○
PWH-8	*Methylobacterium goesingense*	LC896513	99.7	○	○

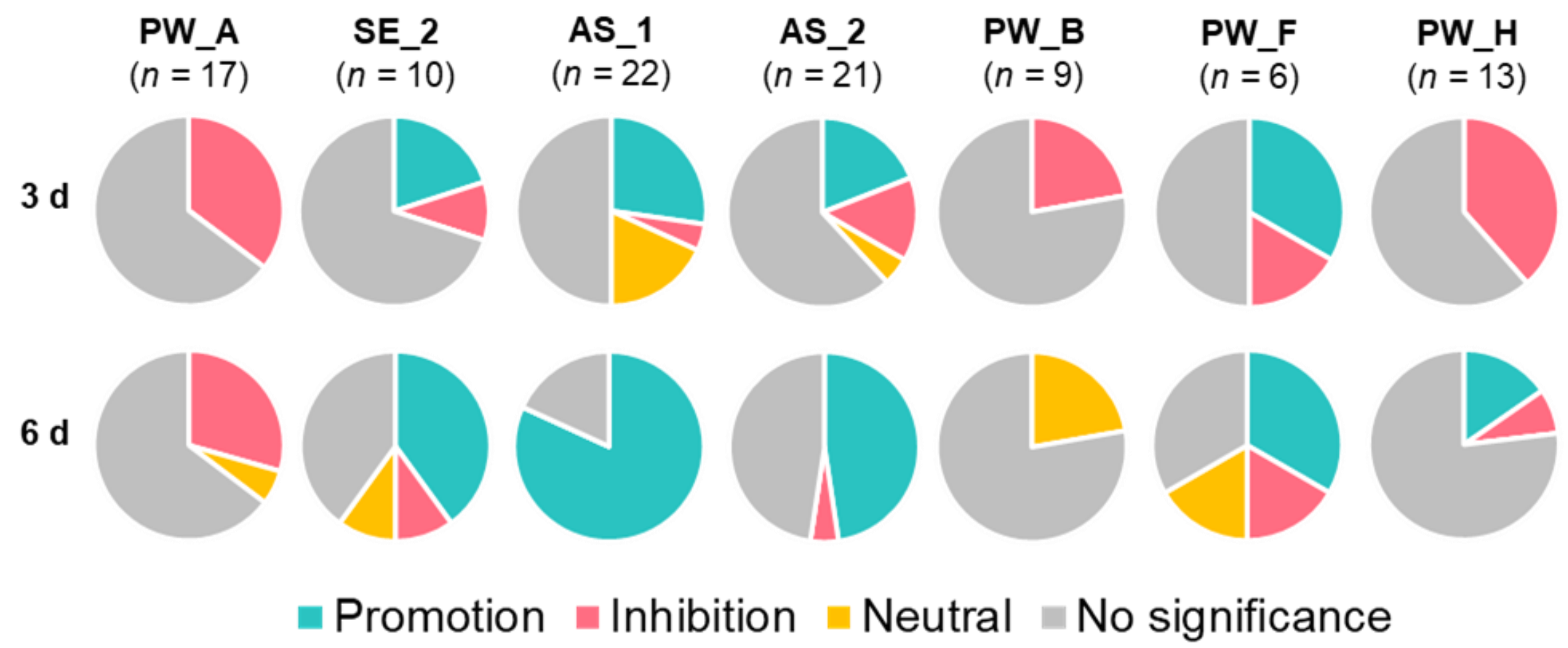

Figure S3. Proportion of isolated strains within each original microbiome showing significant promotive (higher than +5%), neutral (−5% to +5%, but significant effect), and inhibitory effects (lower than −5%), or no significant effect compared with the control after 3 and 6 days.

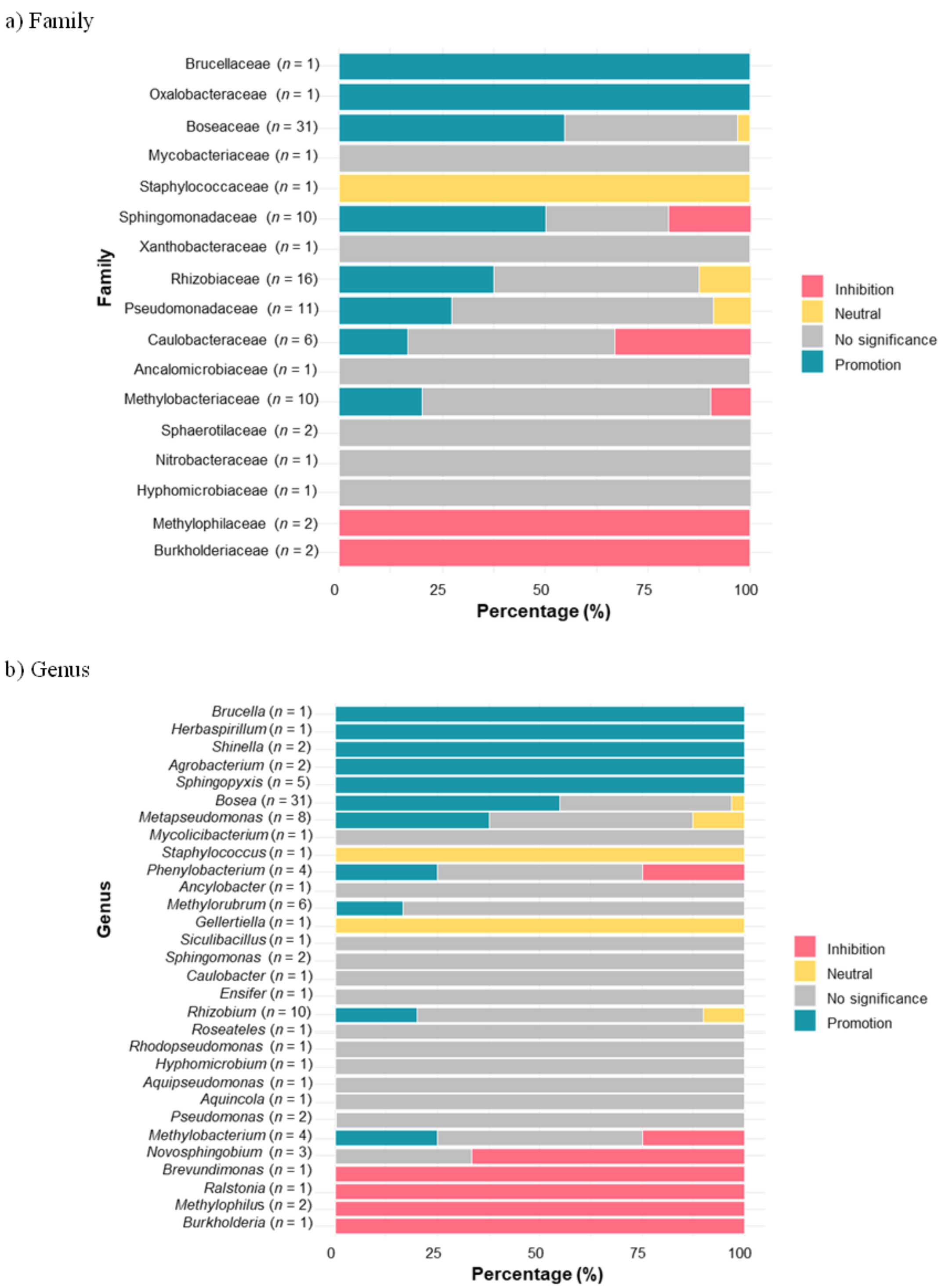

Figure S4. Distribution of the effects of 98 isolated DABs on *Wolffia* growth after 6 days categorized by (a) family and (b) genus.

AIMS Press

© 2026 the Author(s), licensee AIMS Press. This is an open access article distributed under the terms of the Creative Commons Attribution License (https://creativecommons.org/licenses/by/4.0)
